# Current trends and identified gaps in dysphagia research in Africa: A scoping review

**DOI:** 10.4102/sajcd.v72i2.1083

**Published:** 2025-11-18

**Authors:** Skye N. Adams, Kim Coutts

**Affiliations:** 1 Department of Speech Pathology and Audiology, School of Human and Community Development, University of the Witwatersrand, Johannesburg, South Africa

**Keywords:** Africa, dysphagia, scoping review, speech therapy, South Africa

## Abstract

**Background:**

In the context of Africa and other resource-limited settings, there is a necessity to address the unique challenges faced by dysphagic patients and to better understand how healthcare professionals, including speech language pathologists (SLPs), can provide contextually and culturally responsive care given our unique healthcare context.

**Objectives:**

Therefore, this scoping review aims to map and synthesise evidence relating to dysphagia research in Africa to understand trends as well as research and clinical gaps.

**Method:**

An electronic search of CINAHL, Medline, Academic Search Premier, Global Health, PubMed was conducted in May 2024. Peer-reviewed, English-language articles published between 2015 and 2024 were retrieved. A total of 627 articles were screened, and 61 were included in the final review.

**Results:**

Broadly, 60.7% of the articles stemmed from SA with 62.3% being conducted by SLPs. The articles predominantly focused on practice (*n* = 39, 63.9%) but also looked at aspects around prevalence, screening, assessment and teaching and learning. Research on dysphagia in Africa highlights the diverse patient populations affected by the condition and the need for interdisciplinary work.

**Conclusion:**

There is a strong trend towards patient descriptions and the need for describing local data and developing tools that are appropriate for our contexts, but the gaps strongly highlight the need for multidisciplinary involvement in dysphagia, which is not being conducted.

**Contribution:**

This study provides some insight into research being conducted in the African continent, including current trends and areas of future research.

## Introduction

Dysphagia is recognised as a significant global health issue, affecting an estimated 8% of the world’s population (Rajati et al., 2022). The systematic review by Rajati et al. (2022) indicated that Africa has the highest prevalence of oropharyngeal dysphagia with a 64% prevalence rate. This is a significant statistic. Dysphagia (both developmental and acquired) affects 16%–23% of the general population globally, rising to 27% in those over 76 years (Clavé & Shaker, 2015; Lefton-Greif et al., 2024; Smithard, 2016). Dysphagia is heterogeneous and can result from a variety of medical conditions. For South Africa, there is a quadruple burden of disease profile, which depicts the general landscape of the most prevalent health conditions, including communicable diseases, non-communicable diseases, trauma and violence-related injuries as well as maternal and child health aspects (Pillay-van Wyk et al., 2016). These conditions can all result in the development of dysphagia but the lack of prevalence data limits the true understanding of the impact (Mophosho & Lydall, 2023; Said et al., 2023). Similar challenges can be assumed for other African countries. Prevalence data for dysphagia are not available for African countries, although recent studies have shown that the prevalence of dysphagia is similar to the global context, if not higher in African countries (Jayes et al., 2024; Rajati et al., 2022).

Given that the medical sequelae of dysphagia are significant, which can lead to an increase in hospital stay, decreased quality of life for the patient and possibly even death, it must be assessed and managed timeously and effectively (Coutts, 2019). Typically, the speech language therapist (SLT) is the primary medical team member to assess and manage the dysphagia but because of its heterogeneity, it cannot be managed in isolation (Rumbach et al., 2016). How the SLT works within a larger multidisciplinary team (MDT) within the African context is not yet understood and is aimed to be explored within this review. Given the complexities of resource scarcity in the healthcare context in Africa, our decision-making paradigms and practice patterns differ greatly from the Global North, and thus research from these areas cannot be directly transferred to our context; therefore, creating the need for contextualised research to ensure that our practices are relevant and responsive Pillay & Kathard, 2015. This contextual responsiveness is also an aim of this review.

A further contextual complication is that Africa remains the poorest continent globally (Maathai, 2010), which significantly impacts its healthcare system, patients and staffing. On a larger scale, Africa represents 11%–13% of the world’s population and bears a disproportionate 24% of the global disease burden (Azevedo, 2017). As a result, only 3% of the global health workforce is based in Africa, yet they are responsible for serving over 24% of the population (Hollingworth et al., 2023). In South Africa particularly, 82% of the population requires the use of the public healthcare system, yet the majority of healthcare workers work in the private sector (STATSSA, 2018). This imbalance negatively affects the identification and efficient practice of dysphagia management in this context. Therefore, these contextual restraints further support the need for understanding how dysphagia is researched and practiced in this context.

Looking at the International Classification of Functioning (World Health Organization, 2002), eating forms an intricate part of not only an individual’s physiology to sustain life but is also part of their culture and religion. If their ability to swallow has been affected, this has serious and often negative impacts on their daily functioning as members of a family and their community engagement as well as impacts on their caregivers (Solomon & Coutts, 2020). Given the vastness of cultures and religions in Africa and the role that food plays in each of them, this needs to be considered by healthcare practitioners who work in dysphagia. Threats (2007) discusses how the ICF can be used to better understand the impact of dysphagia on the functioning of an individual. Eating and drinking are important activities intrinsically tied to identity and community (Leslie & Lisiecka, 2020). Across societies, people have different understandings and considerations around meal preparation, eating and aspects around ceremonies and dining experiences. An example of this is the cultural manifestations of palliative care and its influence on dysphagia management – particularly post stroke. In many African cultures, there are different reasons a person may refuse to eat which could be linked to spiritual or religious reasons, and this may impact on the ways in which counselling and treatment are being provided, particularly considerations around diet modifications and tube feeding (Grant et al., 2011). Therefore, in a culturally and religiously diverse setting such as Africa, these aspects need to be taught and considered as part of the assessment and management of dysphagia to manage a patient ethically and holistically.

Given this backdrop, there is a necessity to address the unique challenges faced by dysphagic patients and to better understand how SLTs together with the MDT can provide contextually responsive care within our unique and often challenging context (Jayes et al., 2024). Understanding current research trends is critical to developing standardised practices for teaching, assessing and managing dysphagia in Africa. This knowledge can lead to improved outcomes for individuals with dysphagia and contribute to the global understanding of this condition. Therefore, this scoping review aims to map and synthesise evidence of dysphagia research in Africa to understand our current research trends as well as clinical and teaching gaps.

## Research methods and design

A scoping review methodology as suggested by Arksey and O’Malley (2005) was chosen, given the research aim. This framework involves five stages: (1) identifying the research question, (2) identifying relevant studies, (3) study selection, (4) charting the data and (5) collating, summarising and reporting the results used to conduct the scoping review. An ethics waiver was received from the University of the Witwatersrand.

The primary aim was to generate a comprehensive scoping review of research that has been conducted in Africa between 2015 and 2024. The objectives were to determine current clinical as well as teaching and learning trends regarding dysphagia in Africa and to highlight any gaps or areas of future research. The studies that were included in this review needed to meet the following inclusion criteria: published between 2015 and 2024 in accredited peer-reviewed journals, preferably printed in English, studies needed to be conducted in Africa and needed to have a primary focus on dysphagia. Studies could have been conducted by any profession and in any healthcare context.

### Data sources and search strategy

A systematic electronic search for articles was conducted in May 2024 by Dr Skye Adams and Dr Kim Coutts, and all articles until then were included. Six databases were used: CINAHL, Medline, Academic Search Premier, Global Health, PubMed, as well as the first five pages of Google Scholar. The databases were chosen as they were most likely to have publications related to the topic. While Medline and PubMed both provide access to biomedical and health-related research, they differ in scope and indexing. Both databases were included to ensure no relevant records were overlooked, particularly pre-publication articles available on PubMed. To maintain a systematic and replicable approach, the Google Scholar search strategy involved screening the first five pages of results for each search term combination, as research indicates that most relevant and high-quality results are captured within this range. The search strategy, encompassing all identified keywords and index terms, was customised for each database or information source. BOOLEAN operators (AND and OR) as well as truncation were used in the search strings using subject headings (MeSH), and keywords with proximity operators, respectively. The search strategy included subject headings for dysphagia (MeSH) in PubMed and either subject headings for countries and continents or country names. Some articles contained authors from multiple countries or regions within one paper. However, the countries had to be clearly delineated to be included and therefore, it was possible to have an article that had multiple countries or regions.

### Study selection

The current review included published peer-reviewed journal articles, was limited to primary study designs (qualitative, quantitative and mixed-method approaches) and excluded grey literature, other reviews and non-academic sources. Grey literature and non-academic sources were excluded from the study to ensure the use of high-quality, reliable and peer-reviewed academic sources, maintaining focus and reproducibility within the constraints of time and resources.

Following the database searches conducted in June 2024 (SNA), all identified peer-reviewed results were collated and uploaded into Mendeley. All duplicates were removed. The initial search results yielded the following results: PubMed (*n* = 175), Medline (*n* = 126), CINAHL allied and nursing (*n* = 34), Global Health (*n* = 55), Academic Search Premier (*n* = 58), African Journal Archive (139) and Google Scholar articles from the first five pages (*n* = 40). Titles and abstracts were screened by two independent reviewers (K.C. and S.N.A.), with an additional reviewer providing an independent secondary review for assessment against the inclusion and exclusion criteria. Peer-reviewed results that did not meet the inclusion criteria were excluded. Articles were excluded if they did not focus on dysphagia (e.g. articles focused on stroke or nutritional intake only), were not conducted in Africa or were other systematic/scoping review methodologies. Any disagreements that arose between the reviewers were resolved through discussion and input from an independent reviewer until consensus was achieved. An iterative approach to study screening and selection was employed to emphasise a more inclusive final list of studies where after each round of screening, the authors met to provide feedback, and any adjustments were made. All articles were accessed electronically.

### Quality assurance

Quality was assessed using Quality Assessment for Diverse Studies (QuADS) to assess the methodological quality of the included studies whether qualitative, quantitative or mixed designs (Harrison et al., 2021). The QuADS is composed of 13 items for mixed methodology. Each study is scored on every item (0 = not at all; 1 = very slightly; 2 = moderately; and 3 = complete), and the total score was subsequently converted into a percentage. The QuADS was used as it allows for a comparison between the diverse methodologies used by providing a mean score. This method was selected for its reliability and validity when assessing the quality of diverse study designs. The QuADS is also the only tool that can be applied to mixed designs. Both investigators (K.C. and S.N.A.) assessed the quality of the included studies using the QuADS. Any discrepancy was discussed, and a consensus was reached.

The QuADS tool (Online Appendix Table 1-A1) revealed varying levels of quality. The average rating for the articles was 57% with a maximum of 92% and minimum of 18%. The authors noted that perhaps the quality of the articles was slightly lower because of the journal requirements and limited word counts, thus reducing some of the rationales needed in the methodology. Case studies had the lowest scores. Additionally, many studies may have been exploratory in nature, focusing on emerging themes without robust validation of tools or methods. The QuADS tool did not allow for adequate methodological quantification for these types of studies as it is more of a quantitative-focused tool. This also could have contributed to the lower quality outcomes. In the African context, resource limitations, the absence of standardised tools and the reliance on subjective assessments could also contribute to lower QuADS scores. Therefore, all studies were included regardless of their score.

### Charting the data

The data charting template was created by K.C. and S.N.A. The data extracted included specific details about the study objectives to highlight current trends, gaps and future directions. The initial draft of the chart underwent a pilot test using three randomly selected articles. These were reviewed independently by each author, and with the support of an independent reviewer. Following minor revisions based on the pilot test feedback, the finalised chart was then used independently by both reviewers. Agreement between the two reviewers was 90% during the screening process. Any disagreements were resolved by consensus. As a scoping review methodology is iterative, this allowed for an adjustment to be made regarding the inclusion of relevant studies during consensus discussions (Levac et al., 2010).

### Collating, summarising and reporting the results

After extraction, the findings from the included studies were separated, grouped, abstracted and categorised into themes. Independent categorisation based on the study objectives, challenges and supports was conducted by the two reviewers. A content thematic analysis was conducted using Braun and Clark’s framework (Braun & Clarke, 2006). Two reviewers extracted information from each article, grouped and labelled findings, categorised themes and summarised general trends on research in dysphagia in Africa. The broad themes were related to the objectives of the study. The authors developed a code book (*x*) that provided different codes and descriptions, showing the relevant articles and the frequency of each code. Similar codes were then collated and organised into themes and sub-themes. Prominent themes from the reviewers were then selected, relabelled and finalised after a comprehensive review and discussion between both reviewers.

### Ethical considerations

This article followed all ethical standards for research without direct contact with human or animal subjects.

## Results

**FIGURE 1:**
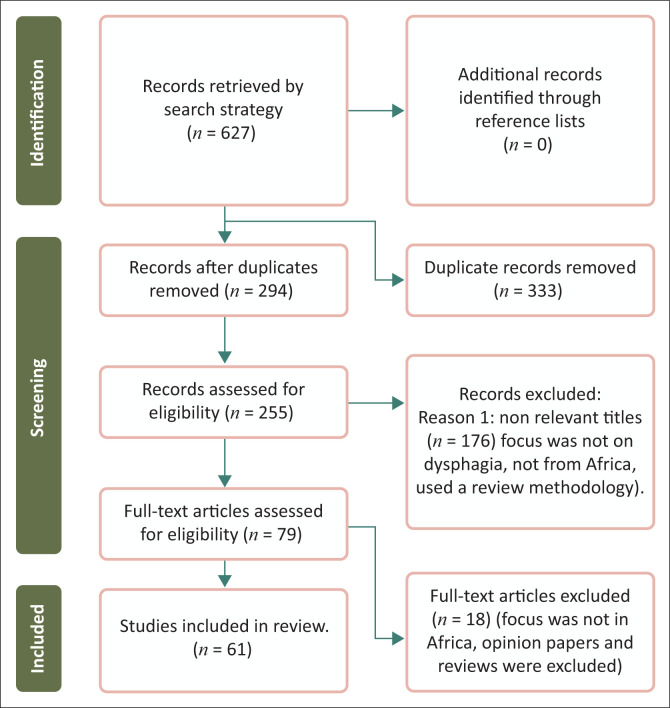
Preferred reporting items for systematic reviews and meta-analysis flow diagram for scoping reviews (PRISMA-ScR).

This review followed the Preferred Reporting Items for Systematic Reviews and Meta-Analyses extension for Scoping Reviews (PRISMA-ScR) (Moher et al., 2009). The initial search yielded 627 articles, and 333 duplicates were removed. No new articles were found through the review of the reference lists. Then, 294 article titles and abstracts were screened for relevance, and 176 were excluded for the following reasons: focus was not on dysphagia, not from Africa, used a review methodology. The full texts of 79 articles were then screened for eligibility, and 61 articles were included in the final review (Figure 1).

### Characteristics of the included studies

**FIGURE 2:**
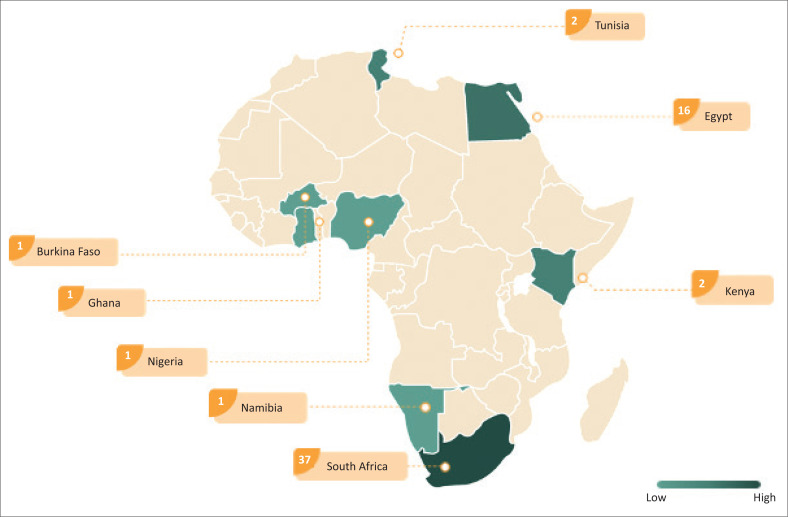
Figure showing countries and number of publications.

A summary of all articles is presented in Table 1. Articles were from seven different countries (see Figure 2). More than half were from South Africa (*n* = 37, 60.7%), whilst others were from Egypt, Namibia, Tunisia, Burkina Faso, Kenya, and Nigeria. South Africa and Egypt appear to contribute the most to dysphagia research in Africa, with other countries contributing less frequently. The majority of studies were written by SLTs (*n* = 38, 62.3%), with the others being written by different specialists in the field of medicine (*n* = 16, 26.2%) and nursing (*n* = 7, 11.5%). Professional distribution regarding multidisciplinary authorship was determined using the first author.

**TABLE 1:** Characteristics of studies identified and included through database research (*N* = 61).

Authors, Year	Country	Design and Methods	Population, Recruitment location, type of treatment choice	Objective	Measures	Results
Abubakar and Jamoh (2017)	Nigeria	**Design:** Correlational Design **Method:** Quantitative	**Sample:** 94 patients **Diagnosis:** Stroke **Adult/Paed:** Adult **Recruitment:** Prospectively enrolled at Ahmadu Bello University Teaching Hospital (ABUTH), Zaria **Discipline:** Medicine	**Prevalence:** To determine the frequency of dysphagia in stroke survivors and its effects on short-term outcome.	**Assessment:** Presence dysphagia: Water-swallowing assessment Stroke severity: National Institute of Health Stroke Scale (NIHSS). Diagnosis pneumonia: presence of three or more variables Outcome measures: 30-day mortality, presence of pneumonia and functional outcome. The functional outcome was assessed at 30 days’ post-stroke using Modified Rankin Scale.	A total of 32 (34.04%) patients had dysphagia at presentation. The mean NIHSS (measure of stroke severity) score was significantly higher in people who had dysphagia compared to those without dysphagia.
Abd Elrhman et al. (2023)	Egypt	**Design:** Cross-sectional descriptive study **Method:** Quantitative	**Sample:** 100 patients **Diagnosis:** Stroke **Adult/Paed**: Adult **Recruitment:** Convenience sampling at Assiut University Hospital **Discipline:** Nursing	**Practice:** To identify risk predictors for post-extubation dysphagia.	**Assessment:** - Demographic and clinical questionnaire - Assessment of the patients’ Hemodynamic parameters - Swallowing ability was screened by Gugging swallowing screen (GUSS). - Direct swallowing test: semisolids, liquids then solids.	More than half of the patients had moderate post-extubation dysphagia. In critically ill patients who have just been extubated, the nurses should screen for dysphagia.
Ali et al. (2022)	Egypt	**Design:** Cross-sectional observational study **Method:** Quantitative	**Sample:** 90 elderly patients **Diagnosis:** N/A **Adult/Paed:** Adult **Recruitment:** Neurological department at Sednawy hospital in Egypt **Discipline:** Nursing	**Prevalence:** To assess the percentage of swallowing disorder among neurological elderly patients.	**Assessment:** Demographic questionnaire Brief Bedside Dysphagia Screening Test – Revised (BBDST-R) – A self-report questionnaire developed by the researcher to assess swallowing in patients with neurological disease	Possible dysphagia in 40% of the studied elderly patients.
Aziz et al. (2023)	Egypt	**Design:** Cross-sectional observational study **Method:** Quantitative	**Sample:** 120 children **Diagnosis:** 60 with neurodevelopmental disorders and 60 controls **Adult/Paed:** Paed **Recruitment:** Children referred to the phoniatrics units of Cairo University and Fayoum University **Discipline:** Medicine	**Practice:** To develop a non-invasive marker to screen for paediatric oropharyngeal dysphagia.	**Assessment** Case history Paediatric Dysphagia Screening Questionnaire (PDSQ) (12 screening questions guided by a comprehensive review of earlier literature and adultscreening tools. Fiberoptic Endoscopic Evaluation of Swallowing (FEES).	Eighty-two per cent of participants in the dysphagia susceptible group had signs suggesting the presence of dysphagia. The PDSQ showed acceptable Sensitivity and Specificity, indicating its validity as a screening tool for pediatric Oropharyngeal Dysphagia.
Andrews and Pillay (2017)	South Africa	**Design:** Survey **Method:** Quantitative	**Sample:** 38 SLP **Diagnosis:** N/A **Adult/Paed:** Adult **Recruitment:** The South African Speech-Language-Hearing Association (SASLHA) recruited by e-mail and via social media **Discipline:** SLP	**Practice:** Explore the clinical practice activities of speech-language therapists in the clinical evaluation of swallowing in adults with acute stroke.	**Electronic questionnaire:** Demographic information Clinical components in the clinical swallow evaluation	SLP used different components when performing a dysphagia assessment that was not standardised. Varying adherence to official policies and fluctuating consistency of clinical practice among.
Bardien et al. (2021)	South Africa	**Design:** Survey **Method:** Quantitative	**Sample:** 26 Nurses **Diagnosis:** N/A **Adult/Paed:** Adult **Recruitment:** Purposive sampling at two acute neurology wards at tertiary hospitals **Discipline:** SLP	**Practice:** Describe barriers to collaborative care and adherence to the SLP’s guidelines perceived by South African nurses.	**Questionnaire** (five sections with a total of 42 questions): Section A focused on knowledge of terminology communication difficulties and dysphagia persons with stroke Section B and Section C focused on the signs and symptoms of communication difficulties and dysphagia Section D related to interpersonal and social system barriers. Open-ended and asked for suggestions for improving nurses’ compliance with SLP recommendations	Nurses had limited understanding of dysphagia and communication disorders but had a good medical understanding of the terminology around dysphagia. Many indicated poor adherence to SLP recommendations because of limited knowledge.
Bhim et al. (2021)	South Africa	**Design:** Retrospective review **Method:** Quantitative	**Sample:** 84 patients **Diagnosis:** Advanced esophageal carcinoma **Adult/Paed:** Adult **Recruitment:** Record review of new patients with oesophageal cancer at the gastro-oesophageal oncology clinic in Cape Town **Discipline:** Medicine	**Practice:** Determine the dysphagia progression-free survival (DPFS) in patients with advanced esophageal carcinoma treated with palliative radiotherapy.	**Assessment:** Radiation therapy – higher dose of RT was offered to patients were planned to use 2-D simulation technique Dysphagia assessment every 6 weeks after completing RT and then routinely every 3 months or earlier if dysphagia worsens. Outcomes: objective worsening of dysphagia – DPFS was taken as the date from the completion of RT to worsening of DS by ≥ 1 point.	Palliative RT can successfully be used to prolong DPFS in patients with locally advanced esophageal cancer.
Blokland et al. (2021)	South Africa	**Design:** Survey **Method:** Quantitative	**Sample:** 60 patients **Diagnosis:** Laryngectomy **Adult/Paed:** Adult **Recruitment**: **Discipline:** Medicine	**Screening:** Develop a 2-question simplified dysphagia score (SDS) screening tool of swallowing function at post-laryngectomy.	**Questionnaire (two sections in English, Afrikaans or Xhosa):** SOAL questionnaire SDS (The questionnaire comprises 5 levels for types of food tolerated, with sub-levels.	The SDS is a 2-question, practical grading system that shows a statistically significant correlation with the SOAL questionnaire, making it a useful alternative for everyday use.
Catania et al. (2023)	South Africa	**Design:** Cross-sectional survey **Method**: Qualitative	**Sample:** 15 SLPs **Diagnosis:** N/A **Adult/Paed:** N/A **Recruitment:** Criterion based purposive sampling UG SLP students at 1 of the 3 South African universities **Discipline:** SLP	**Teaching and learning:** Investigate the factors perceived to enhance critical thinking to shed light on how students transition theory into clinical decision-making.	**Questionnaire (three sections):** Pre-questionnaire to gather information on the participants’ number of clinical hoursand confidence levels in adult dysphagia. 1-min video depicting the FEES procedure on a patient foranalysis. Six open-ended questions regarding intervention for this patient.	To improve transition to clinical in dysphagia students suggested: Videos on instrumental assessment measures, peer collaboration to solve clinical cases, PowerPoint presentations with audio-recordings, exposure to case studies, written explanations and visual illustrations.
Chinniah et al. (2017)	South Africa	**Design:** Case study **Method:** Qualitative	**Sample:** 1 patient **Diagnosis:** Systemic sclerosis (SSC) – myositis overlap and interstitial lung disease **Adult/Paed:** Adult **Recruitment:** Patient at rheumatology department at Inkosi Albert Luthuli Central Hospital in Durban, South Africa **Discipline:** Medicine	**Practice:** Management of a case with systemic sclerosis (SSC) – myositis overlap and interstitial lung disease and treatment of dysphagia.	Clinical assessment	SSC-myositis overlap and severe dysphagia requiring PEG feeding, improved with high dose corticosteroids, azathioprine, two courses of IVIG and rituximab.
Cloete et al. (2022)	South Africa	**Design:** Cross-sectional survey **Method:** Quantitative	**Sample:** 83 SLPs **Diagnosis:** N/A **Adult/Paed:** N/A **Recruitment:** Purposive sampling through social media and online platforms **Discipline:** SLP	**Practice:** Determine the practices and beliefs of SLPs in South Africa regarding tube feeding placement in PWD.	Online questionnaire (32 questions)	SLPs felt they played a role in the decision-making process around tube feeding for patients with dementia. Limited support from MDT. More support required for palliative care.
Solomon and Coutts (2020)	South Africa	**Design:** Cross-sectional interviews **Method:** Qualitative	**Sample:** 7 caregivers **Diagnosis:** N/A (caregivers persons with dysphagia) **Adult/Paed:** Adult **Recruitment:** Purposive sampling from three tertiary public hospitals in an urban area **Discipline:** SLP	**Practice:** Describe the experiences of TPD of caregivers when implementing dysphagia management strategies at home within an economically developing country context.	**Interviews:** Self-developed interview tool that was based on themes of the ICF model domains, resource scarcity and diet modification usage.	The use of diet modification is an appropriate management strategy if the patients’ access and contextual limitations have been taken into consideration.
Coutts and Pillay (2021)	South Africa	**Design:** Observation and focus groups **Method:** Mixed methodology	**Sample:** 7 SLPs **Diagnosis:** N/A **Adult/Paed:** Adult **Recruitment:** Convenience sampling was used to obtain a sample group of seven SLPs who worked at the research site. **Discipline:** SLP	**Practice:** Explore clinical decision making using bedside examinations and presence of dysphagia.	**Assessment:** The Mann Assessment of Swallowing Ability (MASA) – standardised tool that assesses the anatomical and physiological aspects of the patient’s swallow, with different swallowing trials being conducted at the bedside. Observation schedule (aspects around SLP, patient, environmental or contextual and multidisciplinary team (MDT) factors).	Bedside assessment data sets, patient, multidisciplinary team, context influenced clinical bedside decision making. The availability of more data from the assessment from different data sets improved the confidence of the SLP. Clinical instincts are developed through experience and observation of those more experienced.
Coutts and Sayed (2023)	South Africa	**Design:** Cross sectional interviews **Method:** Qualitative	**Sample:** 5 caregivers **Diagnosis:** N/A (caregivers persons with dysphagia) **Adult/Paed:** Adult **Recruitment:** Purposive sampling to obtain the first participant; thereafter, convenience sampling was used to recruit further four adult caregiver participants **Discipline:** SLP	**Practice:** Describe how caregivers experience TPD when caring for adults with dysphagia in Johannesburg.	**Assessment:** Adult Carer-Quality of Life (AC-QoL) Semi-structured interviews (questions focused on support for caring, caring choice, caring stress, money matters and ability to care).	Caregivers experienced challenges related to TPD mostly related to difficulties of being able to do activities of daily living for themselves, their household chores and attending social engagements.
Darwish et al. (2024)	Egypt	**Design:** Cross-sectional observational study **Method:** Quantitative	**Sample:** 200 patients **Diagnosis:** 152 ischaemic, 48 hemorrhagic **Adult/Paed:** Adult **Recruitment:** Purposive sampling from hospital **Discipline:** Medicine	**Prevalence:** To estimate prevalence of dysphagia in first-time hemorrhagic or ischaemic acute stroke patients.	**Assessment:** History and neurology examination Severity of stroke was assessed by using the National Institutes of Health Stroke Scale (NIHSS) Swallowing ability was screened by Gugging swallowing screen (GUSS). Direct swallowing test: semisolids, liquids then solids.	Severity of dysphagia is higher in ischaemic stroke. Old age and severity of stroke were the main determinant of severity of dysphagia.
Diendéré et al. (2018)	Burkina Faso	**Design:** Cross-sectional observational study **Method:** Quantitative	**Sample:** 222 patients **Diagnosis:** N/A (caregivers’ persons with dysphagia) **Adult/Paed:** Adult **Recruitment:** Purposive sampling from hospitals Yalgado Ouédraogo in Ouagadougou and Souro Sanou in Bobo-Dioulasso **Discipline:** Medicine	**Prevalence:** To assess the prevalence of undernourishment and dysphagia in stroke patients in two Burkina Faso teaching hospitals at a starting point (D0), on the eighth day, and on the 14th (D14).	**Assessment:** Sociodemographic data and radioclinical information Weight measurement and BMI Dysphagia identification used the Practical Aspiration Screening Schema system Practical Aspiration Screening Schema test was administered at D0 and D8 and repeated at D14 if clinical notification had been made of dysphagia between D8 and D14.	High prevalence of undernourishment and dysphagia in the stroke patients, of particular concern in a country in dietary transition.
Ebrahim et al. (2018)	Egypt	**Design:** Cross-sectional observational study **Method:** Quantitative	**Sample:** 70 patients **Diagnosis**: Stroke **Adult/Paed:** Adult **Recruitment:** Purposive sampling affiliated university hospital in Cairo **Discipline:** Nursing	**Prevalence:** To explore dysphagia-related health consequences among patients with acute stroke.	**Assessment:** Demographic questionnaire Gugging Swallowing Screen test (GUSS) Direct swallowing test: semisolids, liquids then solids. Post Stroke Pneumonia Assessment tool	There was a significant relationship between degree of dysphagia and (post-stroke pneumonia, death and length of ICU stay). Dysphagia following the acute stroke is very important issue to be considered during handling, caring and management of patients with it.
Ghammam et al. (2019a)	Tunisia	**Design:** Case study **Method:** Qualitative	**Sample:** 2 patients **Diagnosis:** Forestier’s disease **Adult/Paed:** Adult **Recruitment:** Referred to ENT Department and Cervical Surgery Farhat Hached Hospital, Medicine University, Sousse, Tunisia **Discipline:** Medicine	**Practice:** To discuss two cases where the patients have experienced progressive dysphagia.	**Clinical assessment:** Neurological assessment and barium swallow	Forestier’s disease is an uncommon cause of dysphagia mostly affecting older male individuals.
Ghammam et al. (2019b)	Tunisia	**Design:** Case study **Method:** Qualitative	**Sample:** 1 patient **Diagnosis:** N/A (caregivers persons with dysphagia) **Adult/Paed:** Adult **Recruitment:** Referred to ENT Department and Cervical Surgery Farhat Hached Hospital, Medicine University, Sousse, Tunisia **Discipline:** Medicine	**Practice:** To discuss a case of a man who presented with a history of ptyalism and dysphagia occurring after a brain stroke.	Clinical assessment and observation	Retropharyngeal lipoma is one of the exceptional organic aetiologies of dysphagia.
Gomah Yousef et al. (2020)	Egypt	**Design:** Quasi experimental **Method:** Quantitative	**Sample:** 60 patients **Diagnosis:** 30 stroke and 30 control group **Adult/Paed:** Adult **Recruitment:** Purposive sampling at neurological inpatient units at Alexandria University Hospital **Discipline:** Nursing	**Practice:** to determine the effect of swallowing training rehabilitation programme among patients with cerebrovascular stroke.	**Assessment:** Demographic questionnaire Medical data assessment Oral motor structure assessment and reflexes Dysphagia severity scale Observation checklist to assess swallowing ability during swallow trial	Statistically significant difference between the study and control groups in relation to severity of dysphagia and swallowing trial after 2 weeks and 1 month of intervention.
Hady et al. (2023)	Egypt	**Design:** Cross-sectional descriptive study **Method:** Quantitative	**Sample:** 188 healthcare professionals (neurologists, physiotherapists, internal medicine physicians, paediatricians) **Diagnosis:** N/A **Adult/Paed:** N/A **Recruitment:** Convenience sampling of healthcare professionals working in governmental and private hospitals in the Greater Cairo area **Discipline:** Medicine	**Practice:** To determine the level of awareness and knowledge of dysphagia among healthcare professionals.	**Assessment:** Questionnaire divided into four sections: (1) demographic information, (2) awareness and knowledge of dysphagia, (3) practice of dysphagia in the hospitals (including referrals), and (4) level of awareness and knowledge of the SLP role in the management of dysphagia	Minimal awareness was found in specialities with low contact. Fair awareness was found in specialities with moderate to high contact with dysphagia cases. There was insufficient knowledge about non-overt symptoms and signs of dysphagia. Sixty-six per cent of the participants did not receive training in dysphagia.
Hoosain et al. (2024)	South Africa	**Design:** Prospective longitudinal **Method:** Quantitative	**Sample:** 12 children **Diagnosis:** Children on high-flow oxygen **Adult/Paed:** Paed **Recruitment:** Snowball sampling large tertiary hospital in South Africa **Discipline:** SLP	**Practice:** To describe swallowing in children on high-flow oxygen.	**Assessment:** Clinical bedside assessment including follow-up assessment, to track changes in swallowing and feeding characteristics Two assessments were conducted across four food consistencies (liquids, pureed, semi-solid and solid) using the Schedule for Oral Motor Assessment (SOMA)	Oxygen therapy does not preclude children from oral diets. Most participants displayed typical oral motor function at initial and final assessments for liquid, puree and semi-solid consistencies.
Ibrahim et al. (2018)	Egypt	**Design:** Cross sectional descriptive **Method:** Quantitative	**Sample:** 100 geriatric patients **Diagnosis:** Dysphagia **Adult/Paed:** Adult **Recruitment:** Snowball sampling large tertiary hospital in South Africa **Discipline:** Nursing	**Practice:** To identify the factors associated with aspiration risk among geriatric patients with dysphagia.	**Assessment:** Gugging Swallowing Screen (GUSS) Socio-demographic and clinical data of geriatric patients with dysphagia Structured interview schedule (factors associated with aspiration risk among geriatric patients) Barthel Index.	Severe dysphagia with a high risk of aspiration was observed in 37.0% of the study geriatric patients, while moderate dysphagia with a risk of aspiration in 27.0% and those who had slight dysphagia with a low risk of aspiration in 36.0%.
Kater and Seedat (2023)	South Africa	**Design:** Cross-sectional survey **Method:** Quantitative	**Sample:** 16 doctors **Diagnosis:** N/A (Adults with OPD) **Adult/Paed:** Adult **Recruitment:** Non-probability sampling at a medical emergency unit at a public sector hospital in SA **Discipline:** SLP	**Screening:** To establish the reliability and validity of a researcher-developed dysphagia triage checklist.	**Development of the dysphagia triage checklist:** The following dysphagia screening tools were reviewed and adapted: The Standardised Swallow Assessment (Perry, 2001a, 2001b) Massey Bedside Swallowing Screening (Massey & Jedlicka, 2002) Yale Swallow Protocol (Leder & Suiter, 2014) SADS (Ostrofsky & Seedat, 2016).	The checklist was highly sensitive but not reliable or valid for use in identifying patients at risk for dysphagia. Triage for dysphagia took 3 min.
Kaylor and Singh (2023)	South Africa	**Design:** Cross-sectional **Method:** Quantitative	**Sample:** 68 patients **Diagnosis:** StrokeAdult/Paed: Adult **Recruitment:** Convenience sampling at five hospitals, representing four private hospital network groups in the Cape Metropolitan area **Discipline:** SLP	**Practice:** To investigate associations between speech, language, and swallowing conditions (i.e. dysarthria, apraxia of speech, aphasia, dysphagia), and outcomes post-stroke.	**Assessment**: Demographic information Two outcome measure scales were administered by the research personnel at admission and discharge, namely the FOIS and the mRS.	Majority of participants had co-occurring speech, language and swallowing diagnoses, of which dysphagia, aphasia, and dysarthria were the most frequent combination. Participants who were referred to speech therapy later than 24 h post-admission stayed in hospital for longer. Dysphagia was significantly associated with moderate to severe physical disability.
Knight et al. (2020)	South Africa	**Design:** Cross-sectional survey **Method:** Quantitative	**Sample:** 130 nurses **Diagnosis:** N/A **Adult/Paed:** Adult **Recruitment:** Purposive sampling at five sites in a metropolitan area of the Eastern Cape, South Africa **Discipline:** SLP	**Practice:** To describe nurses’ practices related to identification and management of patients with stroke-related OPD.	**Survey (four sections):** Demographic information and level of experience, signs and symptoms, complications and management of dysphagia.	The study found that nurses across all levels of healthcare had only moderate knowledge regarding identification and management of stroke-related OPD.
Kritzinger et al. (2019)	South Africa	**Design:** Cross-sectional observational study **Method:** Quantitative	**Sample:** 81 neonates **Diagnosis:** High-risk neonates **Adult/Paed:** Paed **Recruitment:** Purposive sampling of neonates from neonatal wards of an urban hospital in South Africa **Discipline:** SLP	**Prevalence:** Determine the prevalence and associated risks for OPD in high-risk neonates.	**Assessment:** The Neonatal Feeding Assessment Scale (NFAS) and a pulse oximeter Outcome: presence of OPD	Significant % (64%) neonates presented with OPD and many risk factors.
Krüger et al. (2019)	South Africa	**Design:** Cross-sectional case control **Method:** Quantitative	**Sample:** 28 full-term neonates with HIE and 30 healthy term controls **Diagnosis:** HIE **Adult/Paed:** Paed **Recruitment:** Purposive sampling at (BFHI)-accredited, in Pretoria, South Africa **Discipline:** SLP	**Prevalence:** To identify symptoms of oropharyngeal dysphagia (OPD) in breastfeeding neonates with hypoxicischemic encephalopathy (HIE) on therapeutic hypothermia.	**Assessment:** Demographic information Observation and clinical feeding assessment Breastfeeding behaviour was documented using the Preterm Infant Breastfeeding Behaviour Scale (PIBBS)	HIE babies are at risk for OPD and need to be screened by the MDT.
Krüger et al. (2019)	South Africa	**Design:** Cross sectional prospective **Method:** Quantitative	**Sample:** 71 infants **Diagnosis:** N/A **Adult/Paed:** Paed **Recruitment:** nonprobability purposive sampling neonatal nurseries and postnatal wards of two academic hospitals in Pretoria, South Africa. **Discipline:** SLP	**Practice:** To describe breastfeeding skills of term newborn infants.	**Assessment:** The Preterm Infant Breastfeeding Behaviour Scale (PIBBS) 27 was used to document observable breastfeeding skills Observation before the feeding session. Interview and hospital file review for relevant demographic information and the HIE score	All newborns were exclusively breastfed (link to hospital policy) and presented with typical feeding patterns (some variation noted). HIV exposure did not have significantly different breastfeeding characteristics.
Mahgoub et al. (2024)	Egypt	**Design:** Cross sectional study **Method:** Quantitative	**Sample:** 70 patients **Diagnosis:** Sarcopenia **Recruitment:** Random sampling **Adult/Paed:** Adult **Discipline:** SLP and Medicine	**Practice:** To determine the association between dysphagia and sarcopenia in the elderly.	**Assessment:** Skeletal muscle index, hand grip, the EAT-10 and the Yale swallow protocol	Sixty six per cent of patients had dysphagia, the two dysphagia meausres had good sensitivity and the biggest indicator was low skeletal muscle index.
Malan et al. (2023)	South Africa	**Design:** Longitudinal cohort study **Method:** Quantitative	**Sample:** 29 infants **Diagnosis:** HIE **Adult/Paed:** Paed **Recruitment:** Purposive sampling at a urban tertiary academic hospital in South Africa **Discipline:** SLP	**Practice:** To describe the evolution of swallowing and feeding abilities of neonates with hypoxic-ischaemic encephalopathy (HIE) during hospitalisation.	**Assessment:** Neonatal Feeding Assessment Scale day 1 and on the day each participant was discharged to allow for serial assessment. VFSS prior to discharge from hospital	Approximately two-thirds of participants showed symptoms of oropharyngeal dysphagia (OPD) during initial NFAS and VFSS. Significantly fewer OPD symptoms occurred at discharge. High proportions of participants displayed OPD symptoms regardless of HIE severity.
Masoud Elsaid Hafez and Abo-Baker Mohamed (2023)	Egypt	**Design:** Quasi-experimental **Method:** Quantitative	**Sample**: 60 patients **Diagnosis:** Study group who performed swallowing exercise training, control group who received ordinary routine hospital care **Adult/Paed:** Adult **Recruitment:** Purposive sampling at the Neurological Department at International General Hospital, Mansoura City, Egypt. **Discipline:** Nursing	**Practice:** To evaluate the effect of swallowing exercise training on dysphagia and quality of life among patients following cerebrovascular stroke.	**Assessment:** Demographic information Medical health profile Gugging Swallowing Screen test (GUSS) Direct swallowing test: semisolids, liquids then solids. Functional Oral Intake Scale (FOIS) Quality of Life Scale for stroke patient (QoLSS);	Statistically significant difference in swallowing ability levels and quality of life mean score after implementing swallowing exercise training among patients in study group than control group (*p* < 0.001).
Naidoo et al. (2024)	South Africa	**Design:** Single case **Method:** Qualitative	**Sample:** 4 caregivers **Diagnosis:** N/A (children fed via a long-term non-oral feeding tube) **Adult/Paed:** Paed **Recruitment:** Purposive sampling at one public healthcare hospital in Pietermaritzburg, KwaZulu-Natal. **Discipline:** SLP	**Practice:** identify the significant individuals, factors and views involved in the enteral feeding decision-making process for caregivers of children with CP within the South African public healthcare sector.	**Interviews** (English or isiZulu)	Decision making is paternalistic in nature because of lack of caregiver knowledge and power imbalances.
Ndiema et al. (2024)	Kenya	**Design**: Cross-sectional descriptive design **Method:** Quantitative	**Sample:** 16 healthcare professionals (nurses physiotherapists, SLP, oncologist, neurologist, nutritionist) **Diagnosis:** N/A **Adult/Paed:** Adult **Recruitment:** Purposive sampling at a level-six referral hospital in Nairobi City County, Kenya **Discipline:** SLT	**Practice:** To establish OPD awareness levels among HPs attending to inpatients in the medical and surgical units (acute-care facility).	**Assessment:** Questionnaire that assessed participants’ OPD signs/symptoms awareness levels using 16 items.	Health professionals attending to adult inpatients in the hospital’s medical and surgical units have, on average, moderate awareness of oropharyngeal dysphagia signs and symptoms.
Ndiema et al. (2023)	Kenya	**Design:** Cross-sectional retrospective review **Method:** Quantitative	**Sample:** Thirty-six SLP consultations **Diagnosis:** N/A (Adults with OPD) **Adult/Paed:** Adult **Recruitment:** Patient file review at a hospital in Nairobi City County, Kenya **Discipline:** SLP	**Prevalence:** Establish the demographic characteristics of adult inpatients diagnosed with OPD, determine their OPD-related comorbidities.	58 Patient file reviews	Presentation is high among adults and may vary across specific populations and healthcare settings. Appreciating such variabilities allows HPs to offer holistic and customised care to patients.
Neille and Seliksson (2021)	South Africa	**Design:** Semi structured interviews and observations **Method:** Qualitative	**Sample:** 9 caregivers **Diagnosis:** N/A (children with CP and dysphagia) **Adult/Paed:** Paed **Recruitment:** Purposive sampling at a peri-urban academic hospital in Johannesburg, South Africa **Discipline:** SLP	**Practice:** Explore caregiver factors of moving child onto nonoral feeds and the CDM around it.	**Interviews:** Questions pertaining to the underlying reasons why the child was tube fed, caregiver experiences of transitioning their child to non-oral feeds and the process of decision-making and providing consent, which precipitated the transition, and the support structures available to the caregivers.	Challenges to care and quality of life, access to information and culturally relevant counselling, and the involvement of family members and significant others.
Nel and Omar (2019)	South Africa	**Design:** Comparative cohort study **Method:** Quantitative	**Sample:** 454 patients **Diagnosis:** oesophageal cancer **Recruitment:** Purposive sampling at two hospitals in South Africa **Adult/Paed:** Adult **Discipline:** Medicine	**Assessment:** to assess the profile and management of oesophageal cancer patients at Frere Hospital in the Eastern Cape and compare this to a similar cohort from Groote Schuur Hospital (GSH) in the Western Cape Province.	File review of different data bases analysed on RedCap	Large disparity in medical mx of these cases between two hospitals.
Norman et al. (2024)	South Africa	**Design:** Exploratory study **Method:** Qualitative	**Sample:** 7 parents **Diagnosis:** CHD and dysphagia **Recruitment:** Convenience sampling in a govt hospital in South Africa **Adult/Paed:** Paed **Discipline:** SLP	**Practice:** To describe the burden of care and support needs identified by parents of children with CHD and dysphagia in a single centre in South Africa.	**Semi structured interview:** Questions related to the following main themes: Description of the child’s dysphagia and the impact of dysphagia on the caregiver and family, intervention, support provided to the caregiver with regard to dysphagia and recommendations by the caregiver regarding support that they would have appreciated.	Third party disability was a concern as parents expressed concern around coping, worry, acceptance and need for support from the team. Face-to-face consults were appreciated.
Ostrofsky and Seedat (2016)	South Africa	**Design:** Correlational cross-sectional **Method:** Quantitative	**Sample:** 18 SLPs and 63 stroke patients **Diagnosis:** Stroke **Recruitment:** Convenience sampling in a govt hospital in South Africa **Adult/Paed:** Adult **Discipline:** SLP	**Screening:** To establish the validity and reliability of the South African dysphagia screening tool (SADS) for acute stroke patients.	**Assessment:** SADS Section 1 was aimed at determining the alertness of the stroke participant Section 2: positioning, language, laryngeal functioning, and volitional dry swallow. Section 3: oral motor skills Section 4: food trials	SADS is a valid screening tool that can be used by all healthcare workers. Need tools that can be used by MDT.
Pierpoint and Pillay (2020)	South Africa	**Design:** Descriptive survey **Method:** Quantitative	**Sample:** 21 nurses and 4 doctors **Diagnosis:** N/A **Recruitment:** Non-probability purposive sampling. **Adult/Paed:** Adult **Discipline:** SLP	**Practice:** To explore how doctors and registered nurses, on initial clinical contact, identify and manage post-stroke dysphagia.	**Self-administered paper questionnaire:** Focused on dysphagia risk factors, signs, symptoms, screening and intervention	No formal screenings were used, and nurses and doctors practiced out of scope. MDT did not know dysphagia signs and symptoms. MDT training needs to happen.
Pike et al. (2016)	South Africa	**Design:** 3-group comparative study design **Method:** Quantitative	**Sample:** 49 infants **Diagnosis:** Dysphagia **Recruitment:** Non-probability convenience sampling from govt hospital **Adult/Paed:** Paed **Discipline:** SLP	**Assessment:** To determine whether risk profiles of infants (≥ 32 weeks’ gestation) in a neonatal intensive care unit (NICU) and diagnosed with OPD or OD were distinctly different from one another.	**Assessment:** MBSS, risk assessment checklist and neonatal feeding assessment scale	MDT need to know risk factors that are associated with OPD in order to assist with early identification.
Pillay and Pillay (2023)	South Africa	**Design:** Cross sectional **Method:** Qualitative	**Sample:** 7 SLPs **Diagnosis**: N/A **Recruitment:** purposive, non-random sampling via an email advertisement **Adult/Paed:** N/A **Discipline:** SLP	**Practice:** To explore the nature of clinical reasoning in dysphagia rehabilitation.	Semi-structured interviews with oral histories, cognitive mapping and arts-based tasks	SLPs felt influenced by several manifestations of power within healthcare. We need to transform our approach to clinical reasoning and healthcare by acknowledging and valuing differences in patient and context to allow for truly equitable healthcare provision.
Pullen et al. (2024)	South Africa	**Design:** Cross sectional **Method:** Qualitative	**Sample:** 8 SLPs **Diagnosis:** N/A **Recruitment:** Purposive sampling, online. **Adult/Paed:** Adult **Discipline:** SLP	**Practice:** To describe perceived practices regarding feeding tube placement in people with advanced dementia.	**Self-compiled semi-structured interview schedule:** The questions were tailored according to relevant literature (no details)	Experience is valued in decision making over literature and evidence. We need guidelines and evidence.
Rashed et al. (2023)	Egypt	**Design:** Quasi-experimental design **Method:** Quantitative	**Sample:** 60 children **Diagnosis:** Currently undergoing emergency oral endotracheal intubation **Recruitment:** Purposive sampling at the paediatric intensive care units (PICUs) in emergency hospital **Adult/Paed**: Paed **Discipline:** Nursing	**Practice**: To evaluate the effect of oral care and swallowing interventions on post-extubation dysphagia among children.	**Assessment:** Demographic questionnaire Oral Assessment Guide for Children (OAG) Bazaz dysphagia scale Functional Oral Intake Scale (FOIS)	Nursing intervention that involves swallowing and oral care for a period of 30 min per day for 14th days reduces post-extubation dysphagia, improves clinical swallowing function, and increases the probability of faster oral intake after extubation.
Rhoda et al. (2015)	Namibia	**Design:** Cross sectional survey **Method:** Quantitative	**Sample:** 182 nurses **Diagnosis:** N/A **Recruitment:** Convenience sampling **Adult/Paed:** Adult **Discipline:** SLP	**Practice:** To determine the knowledge and factors associated with knowledge of nurses regarding dysphagia in stroke patients.	Self-developed questionnaire with close-ended questions in two sections: Section A of the instrument included questions relating to socio-demographic characteristics. Section B included questions about the knowledge of the signs and symptoms of dysphagia, complications of dysphagia such as aspiration pneumonia, malnutrition, dehydration, or mortality of stroke patients, and the management of dysphagia in stroke patients.	Nurses have a basic understanding about the signs and symptoms of dysphagia but they need training on the management of dysphagia. MDT practice and training is important.
Robbertse and De Beer (2020)	South Africa	**Design:** Cross sectional survey **Method:** Quantitative	**Sample:** 81 nurses **Diagnosis:** N/A **Recruitment:** Convenience sampling from govt hospitals **Adult/Paed:** Adult **Discipline:** SLP	**Practice:** To determine the specific barriers to compliance with dysphagia recommendations experienced by South African nurses, with the goal of identifying viable strategies to overcome these barriers.	**Survey:** Revised version of the MDQ: 28-Likert Scale responses that range from ‘strongly agree’ to ‘strongly disagree’ on statements regarding knowledge and training-related barriers, patient-related barriers and work-related barriers.	A lack of knowledge regarding dysphagia, patient-related barriers and workplace concerns such as heavy workloads.
Sabry and Abou-Elsaad (2023)	Egypt	**Design:** Comparative study **Method:** Quantitative	**Sample:** 210 patients **Diagnosis:** Stroke **Recruitment:** Sampling not noted (swallows were obtained during standard FEES assessments at Mansoura University Hospital) **Adult/Paed:** Adult **Discipline:** Medicine	**Assessment:** To investigate the correlation between pharyngeal residue with the MFRSS and the PAS on FEES.	**Assessment:** MFRRS, PAS and FEES	There was a high correlation of presence of residue between the two measures and no place of residue was considered riskier.
Schie et al. (2020)	South Africa	**Design:** Retrospective record review **Method:** Quantitative	**Sample:** 4312 patient records **Diagnosis:** N/A **Recruitment:** convenience sampling at govt hospital. **Adult/Paed:** Paed **Discipline:** SLP	**Practice:** To explore feeding and swallowing difficulties in children within the context of South Africa’s quadruple burden of disease.	**Self-developed tool:** Demographic information, admission details, medical diagnoses, BOD classification, referral to SLP, nature of dysphagia (based on clinical bedside evaluations), nature of interventions for dysphagia, mode of nutritional intake at assessment and at discharge, as well as outcomes at discharge that is discharged with no further intervention required, discharged with SLP follow-up or mortality	Paed dysphagia did align with the BoD and children with more complex conditions had dysphagia, need to look at epidemiological factors.
Schoeman and Kritzinger (2017)	South Africa	**Design:** Retrospective record review **Method:** Quantitative	**Sample:** 231 patient records **Diagnosis:** N/A **Recruitment:** convenience sampling at a public hospital for neonates. **Adult/Paed:** Paed **Discipline:** SLP	**Practice:** To describe the feeding characteristics and categories of underlying medical conditions in infants of gestational age 24–42 weeks.	**Record review:** Neonatal discharge report and SLP records	Ninety per cent of babies had multiple medical conditions, 68% had neurological conditions and 65% had subsequent dysphagia.Need to look for risk factors to improve identification of dysphagia.
Seedat and Penn (2016)	South Africa	**Design:** Quasi-experimental parallel group design **Method:** Quantitative	**Sample:** 139 nurses and two groups of participants with oropharyngeal dysphagia. Group one (study group, *n* = 23) Group two (comparison group, *n* = 23) **Diagnosis:** N/A **Recruitment:** convenience sampling at a public hospital **Adult/Paed:** Adult **Discipline:** SLP	**Practice:** to investigate the outcome of an oral care protocol in stroke patients in a govt hospital.	Nurses training on oral care and then record review	Routine oral care is manageable and that a strict routine can reduce aspiration pneumonia.
Seedat and Strime (2022)	South Africa	**Design:** Exploratory study **Method:** Qualitative	**Sample:** 9 nurses Discipline: SLP **Diagnosis:** N/A **Recruitment:** convenience sampling from public hospitals in Johannesburg **Adult/Paed:** N/A **Discipline:** SLP	**Practice:** To determine the level of understanding of dysphagia in nurses in Jhb in state hospitals.	**Semi-structured interviews:** Focused on the frequency of dealing with dysphagia, training, caseload and knowledge and attitudes towards dysphagia	Nurses were not equipped with knowledge and skills for dysphagia and interprofessional training is important.
Sheikhany et al. (2022)	Egypt	**Design:** Cross sectional descriptive study **Method:** Quantitative	**Sample:** 200 elderly patients **Diagnosis:** N/A **Recruitment:** Purposive sampling from a nursing home **Adult/Paed:** Adult **Discipline:** SLP	**Screening:** To determine the use of a screening tool to be used by caregivers to identify dysphagia in the elderly.	**Two self-developed questionnaires:** The dysphagia manifestation one had 14 questions asking about dysphagia manifestations and risk factors such as weight loss, appetite, coughing and changes to eating, Eating habits asked about meal times and consistencies OSME Bedside assessment. Only at risk patients underwent a FEES	The questionnaires were found to be reliable and more screening tools by non-SLPs are important.
Steele-Dadzie et al. (2023)	Ghana	**Design:** Cross sectional descriptive study **Method:** Quantitative	**Sample**: 57 patients **Diagnosis:** Stroke **Recruitment:** Purposive from the Polyclinic, Korle-Bu Teaching Hospital **Adult/Paed:** Adult **Discipline:** Medicine	**Prevalence:** To determine the prevalence of swallowing difficulty among stroke patients and its association with their dietary intake andnutritional risk.	**Assessment** Demographic questionnaire Swallowing difficulty was assessed within 48 h of admission Twenty-four-hour recall interviews of dietary intake Nutritional risk was assessed using the Nutritional Risk Screening	The majority (57.9%) of participants in this study had dysphagia. Dysphagia can lead to a 12-fold increase in developing aspiration pneumonia and subsequent malnutrition.
Visser et al. (2018)	South Africa	**Design:** Two group comparative design **Method:** Quantitative	**Sample:** 12 babies with unrepaired cleft with HIV-E and 13 babies without **Diagnosis:** unrepaired cleft with HIV-E **Recruitment:** Nonprobability purposive sampling at cleft clinic in Pretoria, South Africa **Adult/Paed:** Paed **Discipline:** SLP	**Practice:** to compare the feeding characteristics of babies with an unrepaired cleft with HIV-E and without.	**Assessment:** NFAS and an objective measure (not specified)	Babies with HIV-E present with possible neurological causes for their dysphagia.
Viviers et al. (2019)	South Africa	**Design:** Within subject design **Method:** Quantitative	**Sample:** 48 neonates **Diagnosis:** premature **Recruitment:** Purposive sampling at a public hospital in Pretoria, South Africa **Adult/Paed:** Paed **Discipline:** SLP	**Assessment:** To investigate the reliability and validity of the NFAS in comparison to the MBSS.	**Assessment:** NFAS: six sections including physiological functioning, neonate state, stress cues, motor performance, oral anatomy and signs and symptoms of OPD. MBSS looked at stages of the swallow and indicated if there was the presence of aspiration	Thity one per cent of the babies had OPD, NFAS presented with good reliability and sensitivity to screen for OPD in neonates.
Viviers et al. (2016)	South Africa	**Design:** Comparative within subject design **Method:** Quantitative	**Sample:** 20 neonates **Diagnosis:** N/A **Recruitment:** Purposive sampling at a public hospital in Pretoria, South Africa **Adult/Paed:** Paed **Discipline:** SLP	**Assessment:** To determine the psychometric properties of the NFAS.	**Assessment:** NFAS: six sections including physiological functioning, neonate state, stress cues, motor performance, oral anatomy and signs and symptoms of OPD. MBSS looked at stages of the swallow and indicated if there was the presence of aspiration	A total of 19/20 babies had OPD, 80% of inter-rater agreement and showed good sensitivity (78%).
Viviers et al. (2017)	South Africa	**Design:** Within subject design **Method:** Quantitative	**Sample:** 48 neonates in the NICU **Diagnosis:** N/A **Recruitment:** Purposive sampling at a public hospital in Pretoria, South Africa **Adult/Paed:** Paed **Discipline:** SLP	**Assessment:** To investigate the reliability and validity of the NFAS in comparison to the MBSS in moderate to late preterm infants.	**Assessment:** NFAS: six sections including physiological functioning, neonate state, stress cues, motor performance, oral anatomy and signs and symptoms of OPD. MBSS looked at stages of the swallow and indicated if there was the presence of aspiration. Medical records for medical data and parental interviews were optional (no detail)	Diagnostic agreement between NFAS and MBSS was 85%. NFAS is a reliable tool to identify at-risk neonates. Need to look at test-retest reliability.
Viviers et al. (2020)	South Africa	**Design:** Descriptive comparative group design **Method:** Quantitative	**Sample:** 21 parents **Diagnosis:** N/A (children with autism) **Recruitment:** Purposive sampling online study **Adult/Paed:** Paed **Discipline:** SLP	**Practice:** To describe parent reports of feeding and swallowing difficulties in children with autism between 3–5 years of age.	**Assessment:** BAMBI and a self-developed questionnaire to look at background information such as: demographics, socio-economic factors, biographical information as well as history of feeding and swallowing difficulties	The autism group scored 15% higher in terms of difficulties compared to normally developing group. Swallowing and eating difficulties is multidimensional in nature and the BAMBI can be used as a helpful screening tool for HCWs.
Youssef et al. (2021)	Egypt	**Design:** Cross sectional observational **Method:** Quantitative	**Sample:** 98 patients **Diagnosis:** Dementia and dysphagia **Recruitment:** Non-probability convenience sampling from govt hospital **Adult/Paed:** Adult **Discipline:** Medicine	**Practice:** To determine the risk factors responsible for developing aspiration pneumonia in geriatric patients with dementia and dysphagia.	**Assessment:** Dementia severity was measured with the clinical dementia rating (CDR) scale Assessment of swallowing consisted of a standardised bedside clinical assessment and a Fiberoptic endoscopic evaluation of swallowing (FEES). Penetration aspiration scale (PAS), and pharyngeal stasis scale.	Patients with aspiration pneumonia were of longer hospital stay duration, with more malnutrition, recent stroke, and comorbidities. The risk factors of aspiration pneumonia were severe penetration aspiration, combined tube, and oral feeding, malnutrition, comorbidities, and severe dementia.
Zakaria et al. (2018)	Egypt	**Design:** Cross sectional study **Method:** Quantitative	**Sample:** 200 adult patients **Diagnosis:** Diabetes **Recruitment:** Convenience sampling **Adult/Paed:** Adult **Discipline:** Medicine	**Screening:** To screen for the presence of OPD in adult diabetic patients.	**Screening:** EAT-10	A total of 17.5% of patients presented with dysphagia and DM should be considered a risk factor.
Zayed et al. (2023)	Egypt	**Design:** Descriptive longitudinal study **Method:** Quantitative	**Sample:** 500 adult patients **Diagnosis:** COVID-19 **Recruitment:** Convenience sampling **Adult/Paed:** Adult **Discipline:** Medicine	**Screening:** To screen for OPD in COVID-19 patients. **Discipline:** SLP.	**Assessment:** EAT-10 and Yale swallow protocol	Forty five per cent of patients had OPD and risk factors included age, length of stay, ICU admission, dysphonia, decreased SATS and higher respiratory level and use of mechanical ventilation.

Note: Please see full reference list of this article, Adams, S.N., & Coutts, K. (2025). Current trends and identified gaps in dysphagia research in Africa: A scoping review. *South African Journal of Communication Disorders, 72*(2), a1083. https://doi.org/10.4102/sajcd.v72i2.1083, for more information.

PDSQ, pediatric dysphagia screening questionnaire; SLP, speech language pathologists; TPD, third party disability; PWD, persons with dysphagia; COVID-19, coronavirus disease 2019; ICU, Intensive Care Unit; SATS, Oxygen saturation; RT, radiation therapy; SDS, simplified dysphagia score; BMI, body mass index; SADS, South African dysphagia screening; FOIS, Functional Oral Intake Scale; OPD, oropharyngeal dysphagia; HIE, hypoxicischemic encephalopathy, CDM, clinical decision making; DPFS, dysphagia progression-free survival; N/A, not applicable; Paed, paediatric; SOAL, swallowing outcomes after laryngectomy; PEG, percutaneous endoscopic gastrostomy; IVIG, intravenous immunoglobulin; DS, Dysphagia Score; ENT, Ear, nose and throat; SA, South Africa; mRS, modified Rankin Scale; HP, healthcare professionals; CHD, congenital heart disease; MBSS, Modified Barium Swallow Study; MFRRS, Mansoura Fiberoptic Endoscopic Evaluation of Swallowing Residue Rating Scale; BOD, burden of disease; OSME, Oral Speech Mechanism Examination HIV-E, HIV-Exposed; BAMBI, Brief Autism Mealtime Behavioural Inventory HCW, Health Care Worker; DM, diabetes mellitus.

The methodology types used in the articles were divided into quantitative (*n* = 42, 68.9%), qualitative (*n* = 13, 21.3%) and mixed methods (*n* = 6, 9.8%). The most popular quantitative methods used were survey designs (*n* = 5) and cross-sectional comparative studies (*n* = 10). In terms of the qualitative studies, descriptive studies (*n* = 10) were the most popular. Record reviews were the studies that tended to use mixed method designs. Sample sizes ranged from 1 to 4312, including those with dysphagia, healthcare professional perspectives and patient records.

The proportion of studies that reported subjective outcomes or objective outcomes depended on the specialty of the authors and article aims. The articles predominantly focused on practice (*n* = 39, 63.9%) but also looked at aspects around prevalence (*n* = 8, 13.1), screening (*n* = 6, 9.8%), assessment (*n* = 7, 11.5%) and teaching and learning (*n* = 1, 1.7%). Practice included studies that looked at the perceptions of healthcare professionals and caregivers, the development of tools and guidelines for the management of dysphagia, other medical conditions and the prevalence of dysphagia in certain populations.

### Current trends in dysphagia research in Africa

The most common trends observed in the research related to (1) Participant descriptions, (2) Prevalence studies, (3) Screening and assessment, (4) Practice patterns and (5) Teaching and learning. These are all described in more detail further in the text.

#### Participant descriptions

Participants focused predominantly on adult patients (*n* = 36, 59%) with others including a paediatric (*n* = 16, 26.2%) or a more general dysphagia focus (*n* = 9, 14.8%). Research on dysphagia in Africa highlights the diverse patient populations affected by the condition, including those with cancer, neurological disorders and paediatric feeding difficulties. Eight studies (13.1%) focused on stroke patients with the other studies looking more broadly at patients with varying conditions presenting with dysphagia. Paediatric populations were also of concern, as described by Krüger et al. (2019) and Schie et al. (2020) who examined breastfeeding skills and feeding characteristics of newborn infants and children in South Africa. Across these studies, patient descriptions emphasised the necessity of tailored assessment, interventions and access to care based on specific health conditions and the socioeconomic realities of the African healthcare setting.

#### Prevalence studies

Nine (14.8%) studies explored the prevalence of dysphagia in different patient populations (Abubakar & Jamoh, 2017; Ali et al., 2022; Darwish et al., 2024; Diendéré et al., 2018; Ebrahim et al., 2018; Kritzinger et al., 2019; Ndiema et al., 2023; Schoeman & Kritzinger, 2017). Prevalence was explored in both adult and paediatric populations with several studies looking at the prevalence of oropharyngeal dysphagia in neonates (Kritzinger et al., 2019; Krüger et al., 2019; Schoeman & Kritzinger, 2017). These findings speak to the limited number of studies focusing on prevalence or epidemiology in the African context.

#### Screening and assessment

Effective screening methods for dysphagia are crucial in improving early detection and treatment, particularly in Africa, where resources are often limited. Thirteen (21.3%) of the studies explored different screening or assessments measures (Blokland et al., 2021; Kater & Seedat, 2023; Nel & Omar, 2019; Ostrofsky & Seedat, 2016; Pike et al., 2016; Sheikhany et al., 2022; Viviers et al., 2016, 2017, 2019; Zakaria et al., 2018; Zayed et al., 2023). Studies on screening explored the presence of dysphagia in diabetic patients (Zakaria et al., 2018), stroke patients (Ostrofsky & Seedat, 2016), laryngectomy patients (Blokland et al., 2021), dementia patients (Youssef et al., 2021) and patients with coronavirus disease 2019 (COVID-19) (Zayed et al., 2023). Several studies explored the development or adaptation of assessment and screeners to identify dysphagia and/or swallowing function (Blokland et al., 2021; Kater & Seedat, 2023; Ostrofsky & Seedat, 2016; Sabry & Abou-Elsaad, 2023; Sheikhany et al., 2022), as well as the reliability and validity of certain assessments (Viviers et al., 2016, 2017, 2019). Because of the lack of more objective assessments, studies also looked at the development of non-invasive markers to screen for paediatric oropharyngeal dysphagia (Aziz et al., 2023).

#### Practice patterns

Majority of the studies looked at practice patterns in relation to dysphagia (*n* = 31, 50.8%) (Andrews & Pillay, 2017; Bardien et al., 2021; Bhim et al., 2021; Chinniah & Mody, 2017; Cloete et al., 2022; Coutts & Solomon, 2020; Coutts & Sayed, 2023; Ghammam et al., 2019a, 2019b; Hoosain et al., 2024; Kaylor & Singh, 2023; Knight et al., 2020; Krüger et al., 2019; Malan et al., 2023; Naidoo et al., 2024; Neille & Selikson, 2021; Norman et al., 2024; Pierpoint & Pillay, 2020; Pullen et al., 2024; Rhoda et al., 2015; Robbertse & De Beer, 2020; Schie et al., 2020; Seedat & Penn, 2016; Seedat & Strime, 2022; Solomon & Coutts, 2020; Visser et al., 2018; Viviers et al., 2020; Mahgoub et al., 2024). Practice patterns included the exploration of SLTs practices in evaluating dysphagia (Andrews & Pillay, 2017; Cloete et al., 2022; Seedat & Penn, 2016). Studies also explored the clinical decision-making process around the assessment and management of dysphagia (Coutts & Pillay, 2021).

Practice patterns also included the description of swallowing in different populations (Hoosain et al., 2024; Kaylor & Singh, 2023; Krüger et al., 2019; Malan et al., 2023; Pullen et al., 2024; Schie et al., 2020), working within a collaborative care model with nurses and doctors (Alaraifi et al., 2021; Bardien et al., 2021; Knight et al., 2020; Pierpoint & Pillay, 2020; Rhoda et al., 2015; Robbertse & De Beer, 2020; Seedat & Strime, 2022; Visser et al., 2018). Studies that explored the understanding of dysphagia in professions such as nursing found that nurses tended to have a limited understanding of dysphagia and communication disorders, but had a good medical understanding of the terminology around dysphagia and that nurses and doctors did not always know about signs of dysphagia and the role of the SLT (Hady et al., 2023; Knight et al., 2020; Ndiema et al., 2024; Pierpoint & Pillay, 2020; Rhoda et al., 2015; Robbertse & De Beer, 2020). In addition, there was insufficient knowledge about non-overt symptoms and signs of dysphagia (Hady et al., 2023). All the aforementioned studies highlighted the importance of MDT practice and training and this limited collaborative care.

Practice patterns also included case report studies on how dysphagia was identified/assessed/managed with specific patients (Chinniah & Mody, 2017; Ghammam et al., 2019a, 2019b) and the exploration of third-party disability through caregiver experiences with persons with dysphagia (Coutts & Solomon, 2020; Coutts & Sayed, 2023; Naidoo et al., 2024; Neille & Selikson, 2021; Norman et al., 2024; Solomon & Coutts, 2020; Viviers et al., 2020). Caregiver experiences included parents and other primary caregivers for both adult and paediatric populations. Studies found that all caregivers felt they had a role to play in the decision-making progress around those they care for with dysphagia but were often excluded and required more support from all members of the healthcare team.

#### Teaching and learning

There was only one study that explored dysphagia in relation to teaching and learning (Catania et al., 2023). The study focused on undergraduate SLT students and explored the factors perceived to enhance critical thinking and assist with the transition from theoretical knowledge to clinical practice.

## Discussion

### Research in Africa

The study highlights the current trends in dysphagia research in Africa. Our findings show that there is a paucity of research on dysphagia in the African context given the number of studies that were included in this review. As researchers working in the African context, the authors felt that it is important that SLTs produce research that can be used to extend and challenge existing theory and current practice guidelines, which often stem from the Global North. Despite the opportunity for research in the African setting, there has not been much published research from this context. From 627 articles, only 61 were included for final review. There may be several reasons for the number of articles included in the review, particularly around our current search strategy and the exclusion of grey literature. For instance, it is also important to acknowledge the limitations related to publication and acceptance in mainstream journals. This review is limited to published articles in peer-reviewed journals, and it does not imply that dysphagia research is not occurring in Africa; rather, it suggests that much of this research may not be getting published (Jayes et al., 2024). Many African researchers struggle to publish their findings because of high article processing charges and language and linguistic bias with majority of articles published in Africa coming from English-speaking African countries (Asubiaro & Onaolapo, 2023). Additionally, many journals may not find the research conducted in Africa to be contextually relevant to the Global South where majority of journals originate. Many international journals also have specific requirements that may not be appropriate when conducting research in the African context because of the context, participants and the research infrastructure (Olatunji et al., 2023). As a result, many submissions do not make it beyond the initial editor’s desk review as it often requires changes to comply more with Eurocentric views and western understandings of dysphagia (Draper et al., 2023; Jayes et al., 2024; Klingebiel & Stadler, 2015; Salihu Shinkafi, 2020). The current study highlighted the majority of research coming from South Africa and the inability to generalise findings to the broader African context. Reasons for more research from South Africa could be attributed to better funding, resources and academic support at these institutions in comparison to other African universities (Asubiaro & Onaolapo, 2023). Research from Africa is imperative to scrutinise changes to policy and ensuring findings are contextually and culturally responsive (Kasprowicz et al., 2020). Therefore, this article highlights the need to capacitate research from African researchers in our context and try to establish ways to support and provide appropriate platforms to share research findings. Ensuring that this new knowledge is freely available is imperative for transformation.

### The clinical landscape shaping research, practice and teachings

The clinical context of Africa’s complex healthcare systems has shaped the direction of research, with prominent themes centred on patient descriptors, the development of screening tools and practice patterns. The majority of studies were conducted in South Africa, with other research spanning Egypt, Namibia, Tunisia, Burkina Faso, Kenya, Ghana and Nigeria. These countries have distinct cultural, political, socioeconomic and linguistic landscapes, highlighting the urgent need for research led by African scholars to ensure that practices are contextually responsive. Such research should integrate indigenous knowledge systems to provide a more comprehensive understanding of dysphagia in these diverse contexts (Asubiaro & Onaolapo, 2023; Andrews & Pillay, 2017). Furthermore, these findings highlight the need to establish African-centered indices publication costs, and infrastructure to better support and amplify African research and scholars, particularly to ensure publication of African research in reputable and high-impact journals (Morris et al., 2023).

### The need for interdisciplinary research

While dysphagia is a key area in SLT (Eng & Speyer, 2021), the interdisciplinary nature of this research, involving medicine and nursing, underscores the necessity of context-specific investigations in the African context as seen by multiple studies in this review (Bardien et al., 2021; Knight et al., 2020; Pierpoint & Pillay, 2020; Rhoda et al., 2015; Robbertse & De Beer, 2020; Seedat & Strime, 2022; Visser et al., 2018). Ebrahim et al. (2018) provide a clear example of the importance of interdisciplinary nature in understanding dysphagia-related health consequences in post-stroke pneumonia and the role of the nurses. Nursing staff are critical and were often the focus of research. Nurses support patients by identifying patients who are at risk for swallowing difficulties and managing SLT recommendations around positioning (Rowe et al., 2024). Given that the African context is a resource-constrained setting and that there is a limited number of SLTs, the most effective manner for dysphagia to be screened and identified early is with the use of other members of the MDT.

An MDT approach is important to improve patient outcomes, reduce length and cost of hospital stay and reduce patient load (Atkinson, 2022; Taberna et al., 2020) which are all critical within a resource-constrained setting (Jayes et al., 2024). However, the current review highlighted the lack of MDT support in managing patients with dysphagia and the limited knowledge the nurses had on the scope of SLT and dysphagia assessment and management (Knight et al., 2020; Robbertse & De Beer, 2020). This is significant as interdisciplinary work is not only seen as an advantage for the African context but globally as well (McGinnis et al., 2019) and highlights an opportunity for future research and collaborations across disciplines.

### Research methodologies in dysphagia

Studies utilised different methodologies, with the majority using quantitative methods. This variety is advantageous as the data obtained from varying methodologies have helped to create a comprehensive picture of dysphagia in Africa. Given the significant gaps in our understanding of dysphagia in Africa, there is a need to have a variety of methodologies to address these gaps. Quantitative studies are essential to gain an understanding of patient demographics and assessment tools. However, many of the limitations in the studies were around the small sample size and inability to generalise findings. There was one study by Schie et al. (2020) that had the largest cohort study and good generalisability of findings which was conducted through a record review at a hospital in South Africa. This study highlights the need and opportunities for necessary collaborations for universities and hospitals to be able to work together, particularly within an African context where funds for research and access to participants are often difficult (Klingebiel & Stadler, 2015; Salihu Shinkafi, 2020). Additionally, while prevalence studies are important, research has indicated the importance of using qualitative research methods in an African context. According to Watermeyer and Neille (2022) using qualitative methods can help decolonise research practices by acknowledging and addressing the unique social, cultural and historical contexts of African populations. Given the ICF framework and the diversity of our context, qualitative research is imperative to understand patient experiences and needs, which can then be addressed in dysphagia teachings, practices and policies. This approach is essential for ensuring that research and clinical practices are truly transformative and effective in Africa.

### Clinical tools

The review identified several studies that focused on screening and assessment, which are crucial components in the evaluation of swallowing physiology and the management of dysphagia (Elluru, 2024; Etges et al., 2014; Wilkinson et al., 2021). In Africa, the use of instrumental assessment tools, like videofluoroscopy (VFSS) or fibreoptic endoscopic evaluation of swallowing (FEES), is often challenging because of resource constraints and the fact that clinicians are reliant on more non-instrumental methods of assessment. Therefore, developing and adapting appropriate screening tools is essential. International tools may not always be suitable because of differences in populations, environments and healthcare systems, highlighting the need for contextually relevant assessments (Ostrofsky & Seedat, 2016). This development is crucial to ensure that screening tools are culturally and contextually appropriate, enhancing their effectiveness and reliability in African settings. Research on this needs to continue but access to this research for clinicians needs to be a focus to ensure that practices are contextually responsive.

### Teaching and learning in dysphagia

Alarmingly, there was only one study that focused on aspects around teaching and learning in the field of dysphagia in Africa (Catania et al., 2023). There were also no studies that looked at how we can teach across fields of study, potentially addressing the need for interdisciplinary teaching in dysphagia which could translate into improved interdisciplinary practices. This needs to be addressed. Increasing the knowledge of nursing and other healthcare professionals related to the detection of swallowing difficulties may have a direct impact on patient outcomes (Blackwell & Littljohns, 2010) and should be better utilised in the African context and requires further exploration.

### Limitations of the study

We recognise the need to address the limitations of this review, which was intended as an initial foundation for further research. The current review did not incorporate any grey literature, which may have excluded studies that are not published in peer-reviewed journals such as student theses and service delivery reports, highlighting the need for a more comprehensive review. We also acknowledge the language limitations of including only English articles and the fact that many African countries speak a multitude of languages. These limitations highlight that there may be additional dysphagia researches in Africa that were not included in the current review.

## Conclusion

Dysphagia is prevalent in Africa and is influenced by the unique African socio-economic, cultural diversity and healthcare challenges. Research on dysphagia in Africa remains underfunded and under-represented on a global scale, and the primary source of research in Africa stems from South Africa, which under-represents the continent. Current research trends have largely focused on clinical practice, yet there is a critical gap in studies addressing teaching and learning in this field. This imbalance highlights the urgent need to prioritise educational and interdisciplinary research to enhance capacity-building and improve patient outcomes in this complex setting. Furthermore, addressing these gaps will require a collaborative and interdisciplinary approach, bridging gaps between professions, sectors and disciplines. By fostering partnerships and supporting locally driven research, the field can better respond to the unique realities of dysphagia in Africa, ultimately contributing to more contextually responsive care.
